# Spectrum of Skeletal Imaging Features in Osteopetrosis: Inheritance Pattern and Radiological Associations

**DOI:** 10.3390/genes13111965

**Published:** 2022-10-28

**Authors:** Paolo Spinnato, Elena Pedrini, Miriana Rosaria Petrera, Paola Zarantonello, Giovanni Trisolino, Luca Sangiorgi, Maria Carpenzano, Amandine Crombé, Cecilia Tetta

**Affiliations:** 1 Diagnostic and Interventional Radiology, IRCCS Istituto Ortopedico Rizzoli, 40136 Bologna, Italy; 2 Department of Rare Skeletal Disorders, IRCCS Istituto Ortopedico Rizzoli, 40136 Bologna, Italy; 3 Pediatrics Orthopaedic and Traumatology, IRCCS Istituto Ortopedico Rizzoli, 40136 Bologna, Italy; 4 Department of Musculoskeletal Imaging, Pellegrin University Hospital, FR-33076 Bordeaux, France

**Keywords:** radiography, magnetic resonance imaging, multidetector computed tomography, osteopetrosis

## Abstract

Osteopetrosis (from the Greek “osteo”: bone; “petrosis”: stone) is a clinically and genetically heterogeneous group of rare diseases of the skeleton, sharing the same main characteristic of an abnormally increased bone density. Dense bones in radiological studies are considered the hallmark of these diseases, and the reason for the common term used: “Marble bone disease”. Interestingly, a radiologist, Dr. Albers-Schonberg, described this disease for the first time in Germany in 1904. Indeed, radiology has a key role in the clinical diagnosis of osteopetrosis and is fundamental in assessing the disease severity and complications, as well as in follow-up controls and the evaluation of the response to treatment. Osteopetrosis includes a broad spectrum of genetic mutations with very different clinical symptoms, age onset, and prognosis (from mild to severe). This diversity translates into different imaging patterns related to specific mutations, and different disease severity. The main recognized types of osteopetrosis are the infantile malignant forms with autosomal recessive transmission (ARO—including the rarer X-linked recessive form); the intermediate autosomal recessive form (IAO); and the autosomal dominant ones ADO, type I, and type II, the latter being called ‘Albers-Schonberg’ disease. Imaging features may change among those distinct types with different patterns, severities, skeletal segment involvement, and speeds of progression. There are several classical and well-recognized radiological features related to osteopetrosis: increased bone density (all types with different degrees of severity assuming a ‘Marble Bone Appearance’ especially in the ARO type), different metaphyseal alterations/enlargement including the so-called ‘Erlenmeyer flask deformity’ (particularly of femoral bones, more frequent in ADO type 2, and less frequent in ARO and IAO), ‘bone in bone’ appearance (more frequent in ADO type 2, less frequent in ARO and IAO), and ‘rugger-jersey spine’ appearance (typical of ADO type 2). After conducting an overview of the epidemiological and clinical characteristic of the disease, this review article aims at summarizing the main radiological features found in different forms of osteopetrosis together with their inheritance pattern.

## 1. Introduction

Osteopetrosis refers to a clinically and genetically heterogeneous group of rare, inheritable disorders of the skeleton, characterized by an abnormal increase in bone density on radiographs. For this reason, osteopetrosis is also called ‘marble bone disease’ [1,2]. This condition results from an osteoclast dysfunction or abnormal osteoclast differentiation, leading to a defect in bony resorption. The bone mass appears to be increased and abnormally dense with characteristic bone brittleness.

Osteopetrosis was originally described by the radiologist Dr. Albers-Schonberg in Germany in 1904, even though the term derives from Greek (“osteo”: bone; “petrosis”: stone) [3].

The different osteopetrosis groups also arise from different inheritable conditions, all characterized by a defect in bone resorption, abnormal bone growth and remodelling, osteosclerosis, and higher bony brittleness [4]. The typical radiographic finding is an abnormally increased bone density, resulting in a frequent ‘bone within a bone’ appearance [5,6]. The atypical osteoclast activity also alters the bony shape and structure, modifying its capacity to remodel during growth. In addition, the medullary cavity becomes filled with endochondral new bone, which leaves little space for hematopoietic cells, causing higher bony brittleness [4].

Four main types of disease have been identified based on different models of inheritance and severity [3,4]:

The infantile or ‘malignant’ form: it is an autosomal recessive osteopetrosis (ARO), typically diagnosed within the first year of birth and characterized by a high severity that could lead to mortality in early childhood [6,7]. This form includes the so-called X-linked variant of osteopetrosis, an extremely rare and clinically severe disease, with a recessive transmission such as the other ‘malignant’ form. It can be noted that only a few patients with X-linked type osteopetrosis are reported in the literature.

The ‘intermediate’ autosomal osteopetrosis (IAO): this form is due to an autosomal recessive transmission, which usually becomes clinically significant during the first decade of life. The typical signs are pathologic fractures and progressive cranial nerve compression neuropathies. This form is milder than the previous condition and the affected patients typically reach adulthood.

The autosomal dominant osteopetrosis (ADO), type I: patients are often asymptomatic and, in general, the fracture risk is not increased. The typical sign appears as an isolated osteosclerotic thickening of the cranial vault.

The ADO, type II (Albert-Schönberg disease): even for this form, patients may often be asymptomatic for a long time, but it will eventually present in adulthood with anaemia, pathologic fracture, bone pain, cranial nerve compression, or early arthritis [8].

Mutations involving at least eight genes have been identified as responsible for osteopetrosis pathogenesis in humans [1,9]. Six of these eight genes have been identified in the malignant form (TCIRG1, OSTM1, PLEKHM1, SNX10, TNFSF11, TNFRSF11A), while the mutation of the CLCN7 gene (which codes for the chloride channel 7 protein) has been identified both in ARO and ADO (types I and II). Instead, the IAO results from a loss of function mutation in CAII, the gene responsible for the carbonic anhydrase II protein [9]. Because mutations in the genes described so far only account for approximately 70% of cases, it is highly likely that additional genes involved in the pathogenesis of osteopetrosis will be discovered.

Radiological studies are fundamental for the clinical diagnosis of osteopetrosis. They are able to assess the severity of the disease, usually associated with different known genetic mutations. If typical radiographic signs for osteopetrosis are missing, raised blood concentrations of the creatine kinase BB isoenzyme and tartrate resistant acid phosphatase (TRAP) can be helpful for diagnosis. In addition, genetic testing should be performed to evaluate the presence of a gene mutation, because the distinction between different subtypes is important given the different response to treatments, prognosis, and recurrence risks.

It is important to underline that many other conditions can secondarily or primarily lead to bone sclerosis and should be considered and distinguished from osteopetrosis, particularly: myelofibrosis, hyperparathyroidism, Paget disease (late sclerosing stage), malignancies such as lymphoma or osteoblastic bony metastases, beryllium, lead, and bismuth poisoning, fluorosis, osteopoikilosis, and melorheostosis.

### 1.1. Epidemiology

Epidemiologically, osteopetrosis is a rare condition, but the overall incidence is difficult to estimate. The ARO has an incidence far less than the ADO (1 in 250,000 births compared to 1 in 20,000 births) [1,4,10].

Males and females are equally affected. Higher incidence rates are present in the Middle East, Russia, Sweden, and some specific regions of Costa Rica.

### 1.2. Physiopathology

Osteoclasts are highly specialized cells, which degrade bone mineral and the organic bone matrix. Osteopetrosis is caused by the failure of osteoclast differentiation or dysfunction, which leads to a disruption in the normal bone balanced homeostasis between osteoclast-mediated resorption and osteoblast-mediated deposition. In osteopetrosis, the osteoclasts appear unable to effectively reabsorb bone, causing an unorganized, overly dense bone. Histologically, the affected bone shows empty lacunae with plugged Haversian canals and calcified cartilage. The main trademark findings of osteopetrosis are the lack of a clear zone and ruffled border, which are signs of defective osteoclasts [4].

### 1.3. Clinical Symptoms

Osteopetrosis includes highly heterogeneous conditions varying in severity from asymptomatic to early fatal.

ARO is typically characterized by severe symptoms which may be life threatening within the first months of life. The combination of total bone mass and density increase, and bony encroachment into the medullary space due to the incorrect bone reabsorption by osteoclasts, led to different phenotypic features and symptoms. Modified craniofacial morphology with macrocephaly, frontal bossing, dental abnormalities and skull changes are typical and frequently associated with symptoms related to foramina and bony spaces narrowing, such as choanal stenosis, hydrocephalus, and nerve compression. Blindness, facial palsy, and deafness generally occur [1,4].

Short stature, frailty fracture, and osteomyelitis are frequent, but the most serious complications are related to hypocalcaemia and bone marrow suppression with a consequent haematopoiesis impairment. This results in anaemia, abnormal bruising, bleeding abnormalities, frequent infections, and hepatosplenomegaly. Neuropathic ARO, ARO with renal tubular acidosis, and x-linked ARO are rarer variants with variable degrees of severity [1,4]. In addition to classical symptoms linked with classic ARO osteopetrosis, the X-linked subtype is usually associated with immunodeficiency, localized fluid retention, and tissue swelling caused by lymphedema, as well as hair, skin, nails, and sweat gland abnormalities (ectodermal dysplasia).

Intermediate osteopetrosis, which is rarer, presents autosomal recessive inheritance but has a later onset in childhood and milder symptoms

ADO could be asymptomatic up to late adolescence and adulthood. ADO type 1 is characterized by sclerosis, which mainly affects the skull and, especially, the cranial vault, manifesting with no symptoms, pain, or hearing loss. The risk of fractures is not increased [11].

ADO Type 2 is the most common form and is associated with mild to moderate symptoms. Osteosclerosis typically involves the spine and pelvic bones having multiple fractures, which frequently represent the first manifestations of the disease leading to its diagnosis [12].

## 2. Imaging of Osteopetrosis

The diagnosis of osteopetrosis is clinical, based on history and physical examination, and radiological as well. Imaging plays a useful role in detecting the typical radiological manifestations and identifying sites of skeletal involvement [13]. Indeed, imaging investigations are central to establishing a clinical diagnosis of osteopetrosis [14]. In severe forms, radiological examinations can provide rapid confirmation of the suspected diagnosis. On the contrary, in milder forms, imaging findings may be the first or even the only signs of the disease [15].

Various imaging techniques may be helpful for the assessment of the different features of the disease in the skeleton, and also to evaluate complications.

Conventional radiography alone can show the main well-recognized patterns of osteopetrosis, and above all the abnormal increased bone density.

Computed Tomography (CT) can detect all the patterns of osteopetrosis as well, offering a higher level of detail and higher spatial resolution than conventional radiography. CT could be helpful in better detecting possibly associated fractures or inflammatory diseases of the bone.

Dual-energy X-ray absorptiometry (DXA) is a radiological tool able to measure bone mineral density (BMD) in a quantitative manner. DXA shows the elevation of BMD in patients affected by osteopetrosis due to the pathologically increased bone density [16]. Even for the mildest forms of osteopetrosis, bone density is markedly elevated on DEXA evaluation, with Z-scores usually of +5 standard deviations or greater [15,17]. DXA is particularly useful in the follow up of the disease, as it has the ability to detect and quantify the progression and/or response to treatments [18,19]. Unfortunately, despite the utility of DXA in the general population, in which low bone mineral density is a well-known risk factor for fracture, this tool is not able to accurately predict the risk for fractures in patients with osteopetrosis [20].

Nuclear medicine examinations can be used to assess osteopetrosis. Particularly, 99mTc-sulfur colloid scintigraphy can be performed to detect the bone marrow distribution of the disease [18]. Moreover, 99mTc-MDP and other bone scans can be used to show uptake at multiple fracture sites. SPECT/CT help with accurately localizing, confirming, or excluding any abnormal sites of increased metabolic activity [18].

Magnetic resonance imaging (MRI) is useful in more severe cases to evaluate the amount of remaining bone marrow space. In addition, an MRI of the brain and orbits could be employed to assess the narrowing of central nerve channels, resulting in nerve compression and neuropathic changes.

### 2.1. Pathophysiological Explanations of Skeletal Imaging Findings

The imaging features in all forms of osteopetrosis are explained by the disordered osteoclast function, which is the central biological defect. The pathophysiology of the disease is characterized by an imbalance between osteoblastic bone addition and osteoclastic bone removal, leading to an unopposed increase in bone mass and density [15]. This results in an increase in bone density (considered the hallmark of the disease), an increase in cortical bone volume (hyperostosis), and in severe cases, leads to a loss of medullary cavities, defective bone modelling (undermodelling or undertubulation), bone fragility with an increased risk of fractures, and bone deformities [15].

Osteoclasts also play a key role in the shaping of bones during growth. Normal long bones of healthy subjects are wider at their epiphyseal, joint forming ends, and taper towards their centre to optimise the load-bearing-to-weight ratio [15]. The conversion of a wider metaphysis to a narrower diaphysis (metaphyseal remodelling) is critically dependent on the normal function of osteoclasts; thus, in osteopetrosis, the long bones are ‘undermodelled’, presenting with an elongated transition from wide metaphysis to narrow diaphysis (especially in the distal femur). The trabecular formation of the secondary spongiosa and the osteoclastic resorption on the endosteal surface are critical for the creation of bone marrow spaces; indeed, this space is diminished or absent in severe osteopetrosis forms, and it is associated with consequent bone marrow failure [15].

### 2.2. Pattern of Imaging Features

**Figure 1:**
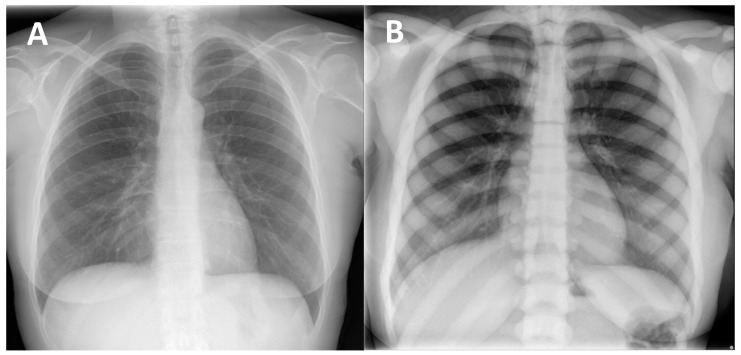
(**A**) conventional radiography of the chest of a young healthy female (shown as comparison). (**B**) conventional radiography of the chest in a 29-year-old female diagnosed with osteopetrosis (clinical and radiological diagnosis in absence of a pathogenic variant in osteopetrosis-related genes). All the skeletal segments included are affected by diffuse and markedly increased bone density (spine, ribs, clavicle, scapulae, humerus): ‘Marble bone’ appearance.

**Figure 2:**
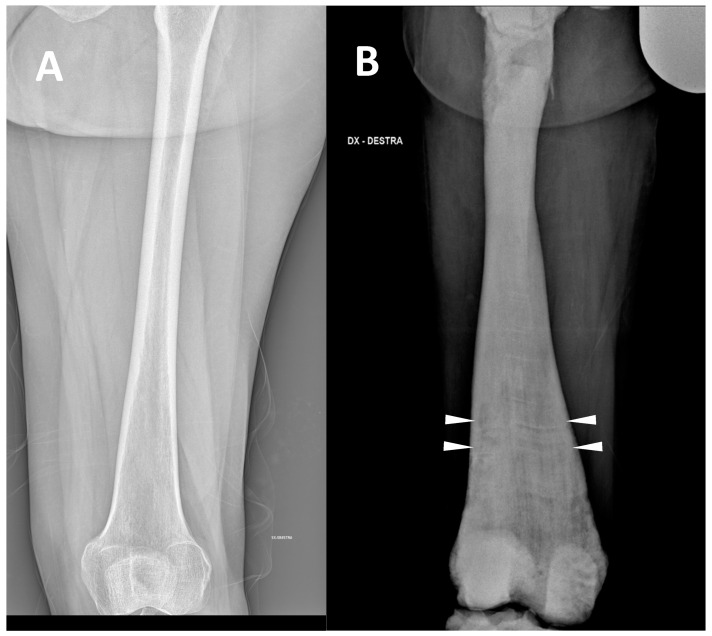
(**A**) conventional radiography of the thigh of a young healthy female (shown as comparison). (**B**) conventional radiography of the right thigh in a 35-year-old male affected by osteopetrosis (ARO type). Metaphyseal enlargement with the so-called ‘Erlenmeyer flask deformity’ of the distal femur, as well as a diffuse increase in bone radiolucency (increased bone density) are detectable. Alternating lucent and sclerotic metaphyseal banding can also be noted.

The most recognized and frequent radiological signs of osteopetrosis can be summarized in four classic imaging features that may appear in the radiological evaluation of patients affected by the disease:

(1) Diffuse increased bone density, sclerosis involving the skull, spine, pelvis, and appendicular bones: This sign is the most known and recognized imaging feature of osteopetrosis, and the reason for the name ‘marble bone disease’ of this condition. Diffuse bone sclerosis can be found in all subtypes of osteopetrosis (ADO, ARO, IAO), even if with slightly different patterns and skeletal locations; particularly in the ARO type, the bone sclerosis can be so marked to assume the so-called ‘marble bone’ appearance (Figure 1).

(2) Defects, enlargement/expansion, and other alterations at long bone metaphysis, which appear widened sometimes with a funnel-like shape (the so-called Erlenmeyer flask deformity) (Figure 2).

This sign consists of a lack of modelling of the dimetaphysis with abnormal cortical thinning and a lack of the concave dimetaphyseal curve [15]. This deformity results in a flask-like appearance of long bones. Indeed, this sign was firstly described in the femur, but it could be similarly found in other long bones such as the tibia and humerus. The metaphyseal enlargement/alteration leading to the ‘Erlenmeyer flask deformity’ appearance can be found in ARO, ADO type 2, and IAO osteopetrosis.

The Erlenmeyer flask deformity can also be found in several other conditions, including multiple exostoses disease, metaphyseal dysplasia, Gaucher’s, Niemann-Pick, and achondroplasia [21,22].

**Figure 3:**
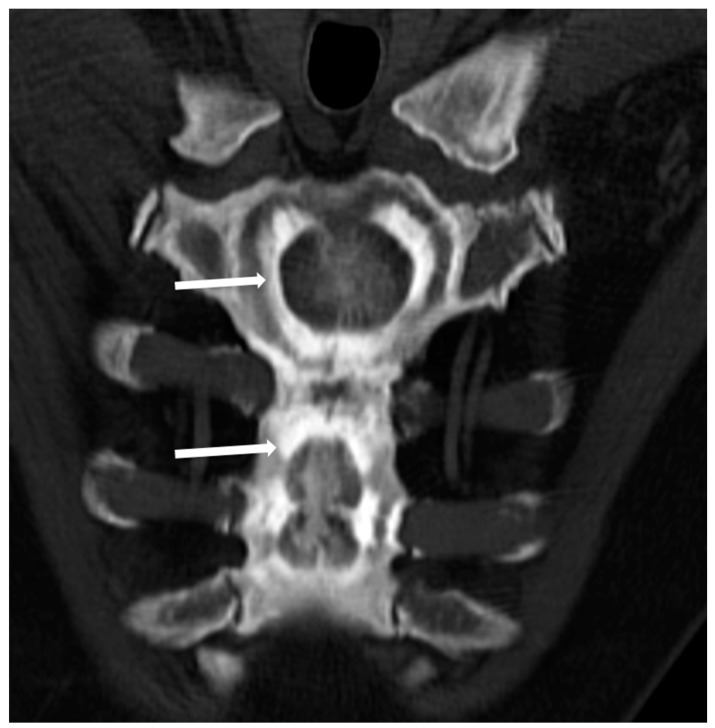
Computed Tomography (CT, coronal-oblique reconstruction) of the sternum in a 48-year-old male affected by osteopetrosis (ADO type 2). The so-called ‘bone in bone’ appearance is detectable with a well-defined increased density within the skeletal segment involved (arrows).

(3) The so-called ‘bone-within-a-bone’ or ‘bone-in-bone’ appearance, characterized by the presence of focal areas of sclerosis within a skeletal segment resembling another bone within the affected bone (Figure 3), which can be mostly found in phalanges, vertebrae, iliac wings, and long bones.

This peculiar imaging feature is more frequently found in ADO type 2 of osteopetrosis, but it can also be encountered in radiological studies of patients affected by ARO and IAO forms.

Several different conditions may lead to the ‘bone in bone’ radiologic appearance in addition to osteopetrosis, including sickle cell anaemia, thalassemia, hypervitaminosis D, rickets, acromegaly, and Gaucher Disease [23,24]. Moreover, it is important to know that this sign is normally encountered in the thoracic and lumbar vertebrae of infants.

**Figure 4:**
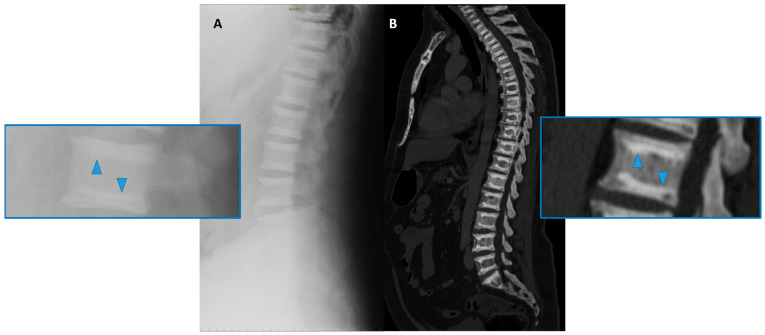
Conventional radiography (lateral projection, (**A**)) and Computed Tomography (CT Sagittal reconstruction, (**B**)) of the spine of a 50-year-old male affected by osteopetrosis (ADO type 2). Well-defined areas of increased bone density are detectable on the vertebral endplates (arrowhead), configuring the so-called ‘sandwich vertebrae’ appearance.

(4) Well-defined sclerosis and the thickening of the vertebral endplate, the so-called “sandwich vertebrae” (Figure 4), and the ill-defined increased density of the vertebral endplates at multiple contiguous levels, the so-called “rugger-jersey” spine, which is, however, more typical of hyperparathyroidism [25,26]; this sign is more frequently encountered in ADO type 2 osteopetrosis.

**Figure 5:**
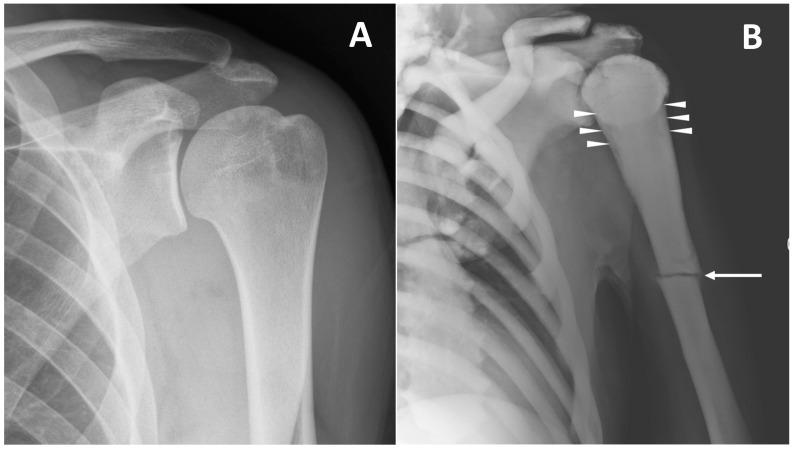
(**A**) conventional radiography of the left shoulder in a young healthy male (shown as comparison). (**B**) conventional radiography of a 45-year-old male with ARO type of osteopetrosis presenting with atraumatic pathological fracture of the diaphysis of the left humerus (arrow). A mild alteration of proximal metaphysis shape (arrowheads), and a diffuse increase in bone density of all the skeletal sites included can also be detected.

Imaging tools are also fundamental for the identification and assessment of complications. Above all complications, pathological bone fractures are the most common ones, and can be found differently in all osteopetrosis subtypes (Figure 5).

**Figure 6:**
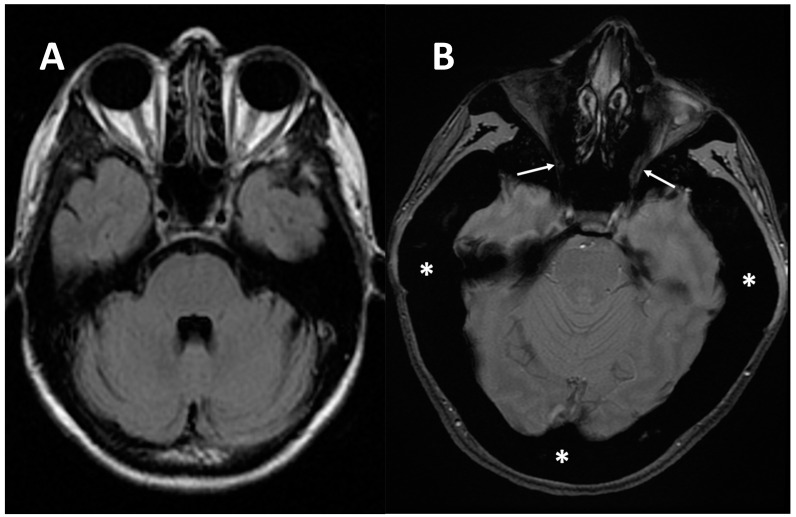
(**A**) axial MRI of a healthy young female (shown as comparison). (**B**) axial MRI (T2w*) of a 38-year-old female with a clinical-radiological diagnosis of osteopetrosis (ARO type); narrowing of both optic foramens can be noted (arrows) caused by a diffuse bone thickening. Diffuse bone thickening of cranial bones is associated with a marked ipointense signal intensity of all the skeletal structures related to the diffuse bone sclerosis (asterisks).

Another common group of complications is neurological involvement, which can be found in patients with ARO or IAO osteopetrosis. Imaging tools, MRIs above all, play a key role in the detection, evaluation, and follow up of neural structures damage. MRI is fundamental to assess cranial foramina (Figure 6) and brain abnormalities.

A relatively less common complication that can be assessed by imaging is osteomyelitis. This complication usually involves the mandible in association with dental abscess and pathological fractures.

**Figure 7:**
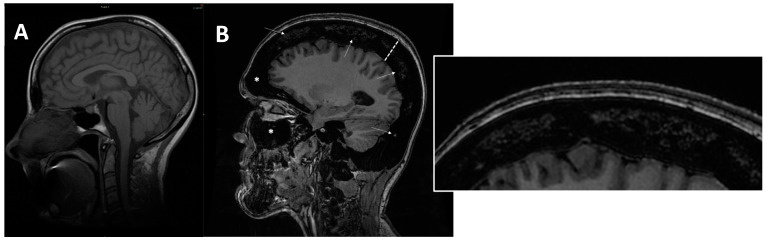
(**A**) axial MRI of a healthy young female (shown as comparison). (**B**) sagittal MRI (proton density sequence) in ARO-type osteopetrosis patient suffering from anaemia (Hb = 7g/dL) shows markedly reduced bone marrow space (arrows and enlargement on the right); diffuse calvarias bone thickening (dotted line), sclerosis (signal hypointensity), and obliteration of maxillary and frontal paranasal sinus (asterisks) can be noted.

Some patients affected by ARO-type osteopetrosis may also suffer from the consequences of reduced bone marrow space (Figure 7), resulting in deficiency of all types of blood cells (pancytopenia), extramedullary haematopoiesis (e.g., in spleen, liver), and can experience the development of myeloid tissue in extramedullary sites (myeloid metaplasia).

The reduced bone marrow space may lead to frequent infections, especially pneumonia and urinary tract infections. Affected individuals may also experience low levels of iron in red blood cells (anaemia) due to both reduced bone marrow space and the increased destruction of red blood cells due to an enlarged spleen. Of note, haematological defects usually present before neurological ones.

Osteopetrosis may affect either the maxilla or mandible, and in these cases, dental radiographs show an increased radiodensity with diminished marrow spaces and thickening of the lamina dura.

### 2.3. Osteopetrosis Inheritance Pattern and Imaging Features Associations

**Figure 8:**
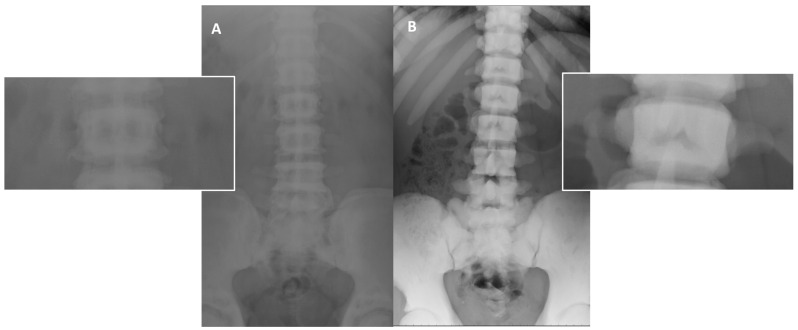
Different pattern of bone sclerosis in different type of osteopetrosis. Conventional radiographies (AP projection) in osteopetrosis ADO type 2 (**A**) and ARO (**B**) show different type of abnormally increased bone density: well-defined sclerosis of vertebral endplate (‘rugger jersey spine’—(**A**)) in ADO type 2, and complete and marked sclerosis of the whole vertebrae in ARO type (**B**).

It is well known that the different genetic patterns of osteopetrosis reflect in different clinical manifestations, prognosis, age of symptoms onset, and nonetheless different radiological features (Figure 8).

As detailed in the cases described, increased bone density was found in all OP types with different degrees of severity assuming a ‘Marble Bone Appearance’, especially in the ARO type, whereby the ‘Erlenmeyer flask deformity’ of the metaphysis of long bones was found particularly on femurs (more frequently in ADO type 2, but also in ARO and IAO), a ‘bone in bone’ appearance was more frequent in ADO type 2 and less frequent in ARO and IAO, and a ‘rugger-jersey spine’ appearance was found in ADO type 2. The thickening and sclerosis of the calvaria or skull base abnormalities are usually found in all osteopetrosis types, even if with different radiological patterns [27]. Indeed, in the ARO type, a diffuse sclerosis of the skull is usually found, together with a thickening of the skull base and calvaria. In the ARO osteopetrosis type, alterations of the facial bones can be found too; particularly hypertelorism, with a so-called ‘space alien’ appearance on the frontal radiographs described in children. Poor pneumatization or the complete obliteration of the paranasal sinuses can be detected on imaging studies of patients with ARO-type osteopetrosis (see Figure 7) [28].

Table 1 summarizes the different types of osteopetrosis, the known genes involved, and their main radiological features together with the associated clinical manifestations.

Nonetheless, some patients with a radiological and clinical diagnosis of osteopetrosis may be negative for all the available genetic tests, suggesting that some mutations remain to be discovered [29]. In these cases, radiology plays a key role in the diagnosis and disease severity stratification without the confirmation of the genetic tests currently available. Moreover, the association of specific radiological features with a specific inheritance pattern is important to facilitate the identification of new genetic determinants responsible for OP.

## 3. Conclusions

Radiology is fundamental in the clinical diagnosis of osteopetrosis since it is able to detect the hallmark of the disease, represented by the increased density of the affected skeletal segments. Moreover, imaging tools are of importance in assessing disease severity and complications, as well as in follow-up controls and treatment-response evaluations.

**Figure 9:**
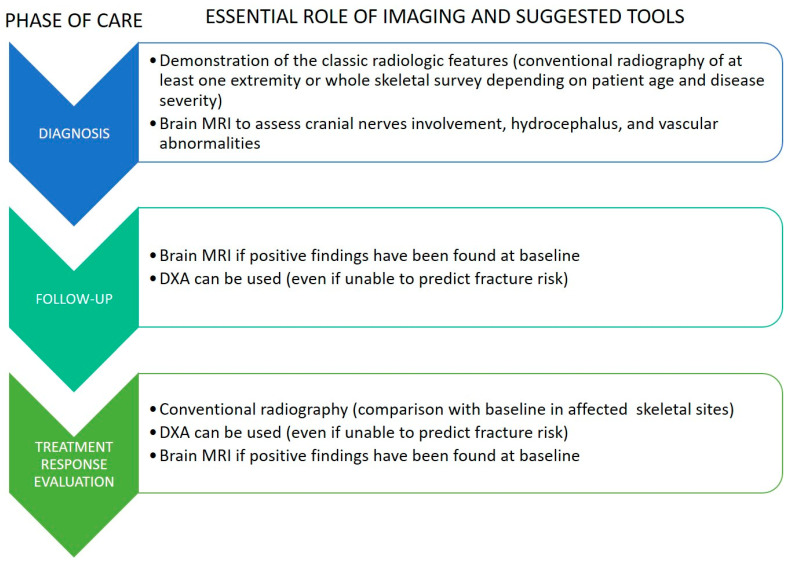
A general flow chart with a summary of essential imaging at each phase of osteopetrosis care.

In Figure 9, a flow-chart with a summary of essential imaging at each phase of osteopetrosis care is presented.

Osteopetrosis includes a wide spectrum of genetic mutations with very different clinical symptoms, age onset, and prognosis (from mild to severe). This is reflected in different imaging patterns related to specific inheritance transmission and disease severity; while some radiological aspects are in common with dominant and recessive forms of osteopetrosis (e.g., diffuse bone sclerosis, ‘bone-in-bone’ appearance), some of them are related to the specific inheritance pattern and, in turn, are related to the disease severity: the ‘marble bone’ appearance seems to be peculiar for the ARO type, the ‘sandwich vertebrae’ are typical of ADO type 2 osteopetrosis, and the ‘bone-in bone’ appearance and ‘rugger-jersey spine’ are more frequently found in ADO type 2. The imaging ability to discriminate some specific genetic and clinical subtypes makes it important in both osteopetrosis diagnosis and treatment—especially in the absence of genetic confirmation. As is known, most osteopetrosis forms can be treated with Haematopoietic Stem Cell Transplantation (HSCT); despite this, in the presence of neurodegenerative forms, as well as when osteopetrosis is caused by mutations in the TNFSF11 gene, the treatment is contraindicated. To date, all the patients with OSTM1 alterations and half of those carrying homozygous CLCN7 mutations are related to neurodegenerative symptoms, thus making essential the molecular diagnosis, especially when the neurological damage may not yet be evident. Unfortunately, the presence of patients lacking a pathologic variant—probably due to the involvement of genes not yet known—and the clinical heterogeneity associated with some genes (CLCN7) makes it mandatory to always perform a careful neurological evaluation of the patient, as this provides a key role for the imaging tools in osteopetrosis disease.

For these reasons, radiologists and specialized clinicians should know and should be able to recognize the characteristics of imaging features in order to help with the proper clinical diagnosis, assess the radiological disease severity, evaluate its progression on follow-up controls, and provide support for research in the molecular diagnostic field by suggesting the presence of a specific inheritance model to help the identification of new genes. Further studies to correlate radiographic and clinical features with the genetic variants will be carried out considering a necessarily higher number of patients; in addition to providing new information on all the molecular pathways involved in osteopetrosis, they will also allow researchers to explain all of the ‘atypical’ osteopetrosis patients, as well as provide new diagnostic tools.

## Figures and Tables

**Table 1:** Different types of osteopetrosis, the known genes involved, and their main radiological features together with the associated clinical manifestations.

Osteopetrosis Type			Imaging Features			Main Clinical Findings
		Genes Involved	Skull	Spine	Appendicular Skeletal	
ARO (autosomal recessive osteopetrosis)	Classic	TCIRG1, CLCN7, less frequently OSTM1, SNX10, TNFRSF11A, TNFSF11	- Diffuse sclerosis - (base + vault thickening)	- Diffuse sclerosis and thickening - Increased bone density	- Diffuse sclerosis and thickening - Increased bone density “marble bone” - Different kind of long bone with metaphyseal enlargement/alterations (possibly with ‘Erlenmeyer flask deformity’ appearance)	- Macrocephaly - Cranial nerve palsies - Hydrocephalus - Optic nerve atrophy - Intrapetrous carotid artery stenosis - Dental abnormalities - Pancytopenia - Hypocalcaemia - Short stature - Fractures - Hepatosplenomegaly for extramedullary haematopoiesis
	Neuropathic		As classic ARO + neurodegeneration MRI findings: (delayed myelinisation cerebral atrophy)	As classic ARO	As classic ARO	- Retinal atrophy - Deafness - Hypotonia
	With renal tubular acidosis		As classic ARO + Cerebral calcifications	As classic ARO	As classic ARO	- Renal tubular acidosis
	X-linked		- Diffuse sclerosis and thickening - Increased bone density	- Diffuse sclerosis and thickening - Increased bone density	- Diffuse sclerosis and thickening - Increased bone density	- Lymphedema - Ectodermal dysplasia - Immunodeficiency
IAO (Intermediate autosomal osteopetrosis)		CLCN7 (dominant or recessive), TCIRG1 (recessive), SNX10 (recessive), TNFRSF11A (recessive), TNFSF11 (recessive), CA2 (recessive), PLEKHM1 (dominant or recessive)	- Diffuse sclerosis and thickening - Increased bone density	- Different degrees of diffuse and focal sclerosis and thickening - Increased bone density	- Different degrees of diffuse and focal sclerosis and thickening - Increased bone density	- Milder symptoms than classic ARO - Pathologic fractures - Nerve compression
ADO (Autosomal dominant osteopetrosis)	ADO type I	CLCN7	- Diffuse sclerosis - (>vault thickening)	Diffuse sclerosis of spine and pelvis	Diffuse thickening	- Asymptomatic - Rare fractures - Bone pain
	ADO type 2 *Albers-Schönberg disease*		- Diffuse sclerosis - (>base thickening)	- Hyperostotic vertebral endplates (sandwich vertebrae and rugger-jersey spine) - “bone in bone” appearance	- Diffuse or focal sclerosis and thickening - Defects at long bone metaphysis with funnel-like appearance (Erlenmeyer flask deformity) - Alternating radiolucent and osteosclerosis bands	- Asymptomatic - Multiple fractures - Scoliosis - Hip osteoarthritis - Osteomyelitis - Back pain - Rare (5% cases) nerve compression with hearing and visual loss

## Data Availability

Not applicable.
